# Global study of anti-NMDA encephalitis: a bibliometric analysis from 2005 to 2023

**DOI:** 10.3389/fneur.2024.1387260

**Published:** 2024-04-22

**Authors:** Xinyue Song, Zixin Luo, Duoqin Huang, Jialian Lv, Li Xiao, Ting Liang, Kang Zou

**Affiliations:** ^1^Gannan Medical University, Ganzhou, Jiangxi, China; ^2^Department of Rehabilitation Medicine, The First Affiliated Hospital of Gannan Medical University, Ganzhou, Jiangxi, China; ^3^Department of Critical Care Medicine, The Second People's Hospital of Nankang District, Ganzhou, Jiangxi, China; ^4^Department of Critical Care Medicine, The First Affiliated Hospital of Gannan Medical University, Ganzhou, Jiangxi, China

**Keywords:** autoimmune encephalitis, anti-NMDA encephalitis, antibodies, research hotspots, bibliometric analysis

## Abstract

**Background:**

Autoimmune diseases have always been one of the difficult diseases of clinical concern. Because of the diversity and complexity of its causative factors, unclear occurrence and development process and difficult treatment, it has become a key disease for researchers to study. And the disease explored in this paper, anti-NMDA encephalitis, belongs to a common type of autoimmune encephalitis. However, the quality of articles and research hotspots in this field are not yet known. Therefore, in this field, we completed a bibliometric and visualization analysis from 2005 to 2023 in order to understand the research hotspots and directions of development in this field.

**Materials and methods:**

We searched the SCI-expanded databases using Web of Science’s core databases on January 22, 2024 and used tools such as VOS viewer, Cite Space, and R software to visualize and analyze the authors, countries, journals, institutions, and keywords of the articles.

**Results:**

A total of 1,161 literatures were retrieved and analyzed in this study. China was the country with the most total publications, and USA and Spain were the most influential countries in the field of anti-NMDA encephalitis. University of Pennsylvania from USA was the institution with the highest number of publications. While Dalmau Josep is the most prolific, influential and contributing author who published one of the most cited articles in Lancet Neurology, which laid the foundation for anti-NMDA encephalitis research, the top three appearances of keyword analysis were: “antibodies”, “diagnosis”, and “autoimmune encephalitis.”

**Conclusion:**

Bibliometric analysis shows that the number of studies on anti-NMDA encephalitis is generally increasing year by year, and it is a hot disease pursued by researchers. USA and Spain are leading in the field of anti-NMDA encephalitis, while China should continue to improve the quality of its own research. The suspected causes of anti-NMDA encephalitis other than ovarian teratoma and herpes simplex, the specific clinical manifestations that are not masked by psychiatric symptoms, the diagnostic modalities that are faster and more accurate than antibody tests, and the improvement of treatment modalities by evaluating prognosis of various types of patients are the hotspots for future research.

## Introduction

Research on antibody (Ab)-mediated encephalitis has progressed tremendously since the discovery that antibodies against the N-methyl-D-aspartate receptor (NMDAR) are associated with a distinct neuropsychiatric syndrome (1). There are many types of autoimmune encephalitis, but anti-NMDA encephalitis is among the most common (2), and commonly affects females, but all ages and genders may be affected. Annually, 1.5 cases are reported per million population, and the impact on neurology and psychiatry is significant (3). To date, many patients have been diagnosed with anti-NMDA-R encephalitis, but the exact prevalence of this disease is unknown (4). Early diagnosis and early treatment can have a dramatic impact on the prognosis of patients with anti-NMDA encephalitis (5). Due to the severity, difficulty of treatment and diversity of occurrence and development of anti-NMDA encephalitis, it can be a great threat to human health, so scholars in this field pay great attention to it.

Bibliometrics is a way to analyze research hotspots and trends in historical classic articles (6). Based on the literature in the database, it can conveniently analyze the distribution structure, quantitative relationship, and changing law of publications. It can comprehensively analyze the information of authors, journals, institutions, countries as well as collaborative networks, keywords, etc. of the articles, and provide readers with the historical development as well as the future trend of the research in this field. Bibliometric analysis is widely used in a variety of research fields today. However, there are few bibliometric studies in the field of anti-NMDA encephalitis, and the research hotspots and trends are not clear. Therefore, this study focuses on the research directions and research hotspots of anti-NMDA encephalitis and analyzes its future research trends.

## Data and methods

### Data sources and search strategies

**Figure 1:**
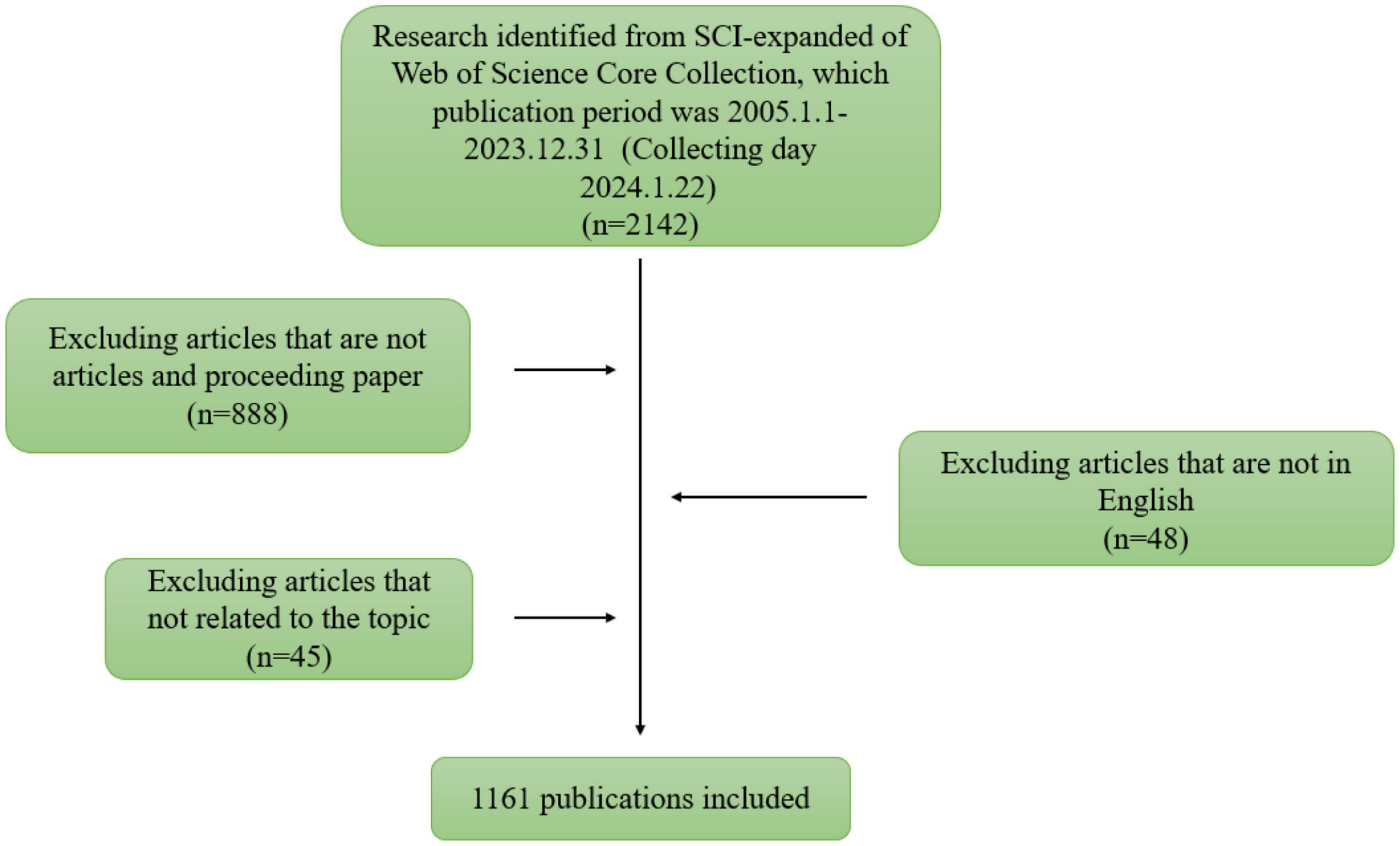
Procedure of the search technique.

We used the Science Citation Index Expanded (developed by Thomson Scientific) database from the Web of Science core databases as the data source for data collection and data analysis. All data collection activities were completed on January 22, 2024 to avoid time bias. The keywords searched were (((((((((TI = (anti-N-Methyl-D-Aspartate Receptor Antibodies encephalitis)) OR TI = (Anti-NMDA-receptor encephalitis)) OR TI = (N-methyl-d-aspartate antibody encephalitis)) OR TI = (Autoimmune N-Methyl-D-Aspartate Receptor Encephalitis)) OR TI = (anti-NMDAR encephalitis)) OR AB = (anti-NMDAR encephalitis)) OR AB = (Autoimmune N-Methyl-D-Aspartate Receptor Encephalitis)) OR AB = (N-methyl-d-aspartate antibody encephalitis)) OR AB = (Anti-NMDA-receptor encephalitis)) OR AB = (anti-N-Methyl-D-Aspartate Receptor Antibodies encephalitis). The publication period was set from January 01, 2005 to December 31, 2023. Publication type was set to article and proceeding paper. Publication language was set to English. The two researchers performed data search and screening separately, excluding articles whose search terms did not appear in the title, abstract, or author keywords. Controversial articles were also discussed, with 99% screening agreement between the two authors. For articles where agreement could not be reached, we consulted a third author. Ultimately, we identified 1,161 articles in the field of anti-NMDA encephalitis for inclusion in the analysis from the above search. They came from 68 countries, 1,646 institutions, 347 journals, and were written by 6,030 authors. Figure 1 shows the above search process in detail.

### Data analysis and visualization

After identifying all the articles analyzed, we exported the articles in TXT format. The exported information was the complete record and references (including title, keywords, authors, institution, country, source, references, etc.). Subsequently, we used Cite Space 6.2. R4 (Chaomei Chen, Drexel University, Dalian, China) (7), VOS viewer (Van Eck and Waltman, The Centre for Science and Technology Studies, Netherlands) (8) and bibliometric (9) in R 4.3.1 software (Robert Gentleman and Ross Ihaka, University of Auckland, New Zealand) to automatically transform and analyze the literature information (8). Information such as title, author, journal, institution, country and region, citation frequency, year of publication, impact factor, and keywords were comprehensively analyzed, integrated and summarized (10).

Impact factor (IF) is an important indicator for evaluating the impact of a journal or literature (11). It can provide readers with a more visual reference for understanding the importance of a journal or literature in the field. Of course, using IF alone to express a researcher’s contribution is not comprehensive enough. Similarly, the number of publications (NP) and the number of no self-citation (NC) of authors are important objective ways of evaluating impact in the field. These factors will be used as evaluation metrics in our analysis of all articles in the material (12).

## Results

### Analysis of annual number of publications and average annual citation frequency

**Figure 2:**
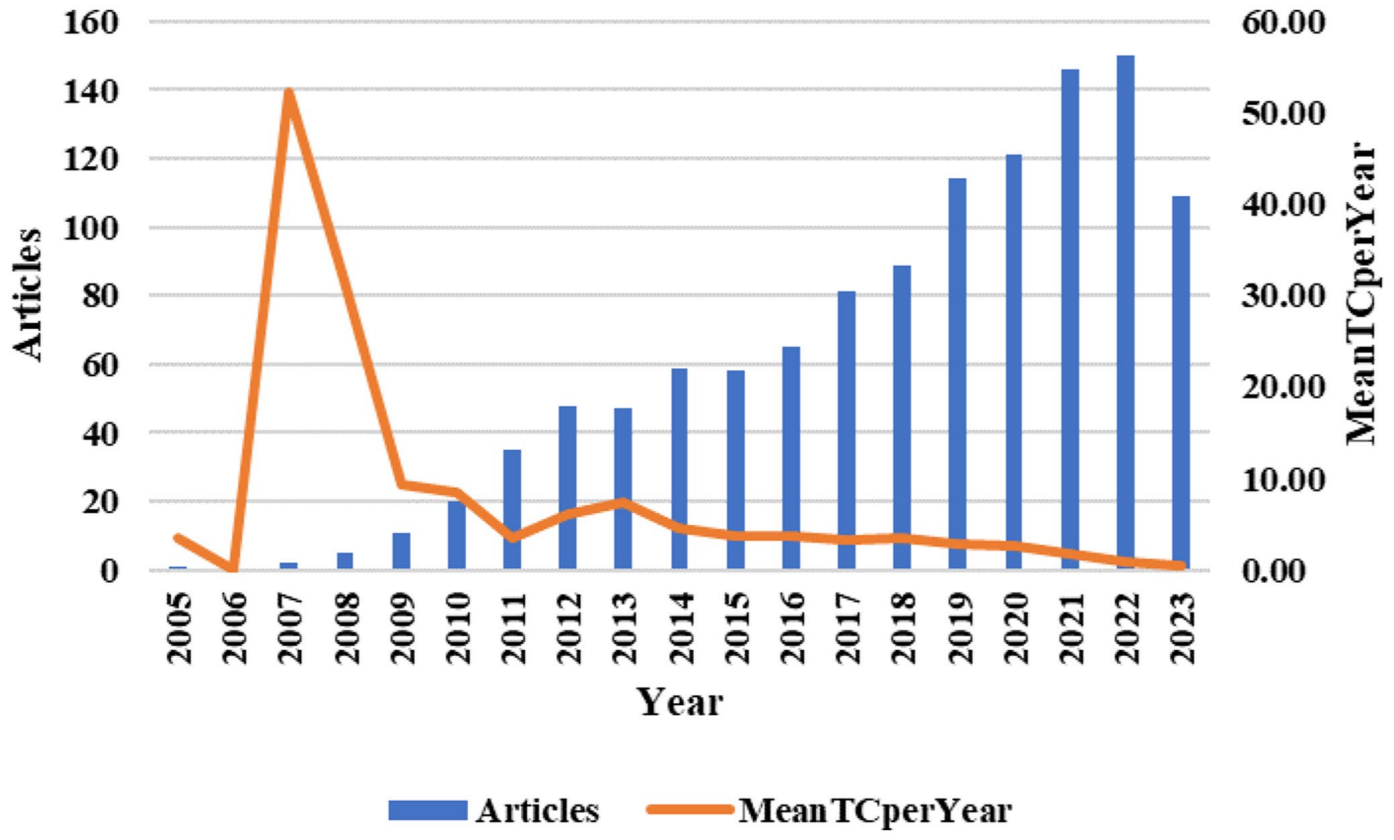
2005–2023 yearly distribution of the number of citations and articles issued of the articles in the ALI/ARDS field. Mean TC per Year, mean total citations per years.

The bar chart shows the number of annual publications issued (represented by the left vertical axis), and the line graph shows the average number of citations per year (represented by the right vertical axis), with the year as the horizontal coordinate (Figure 2). Overall, the number of publications from 2005 to 2021 showed an increasing trend with the year, rising to 150 and peaking in 2022. 2018 (*n* = 89) to 2019 (*n* = 114) showed the largest increase of 21.93%. While from 2022 to 2023 there is a decreasing tendency with a larger decrease of only 109 articles in 2023. The average number of citations was highest in 2007, and the rest of the years were generally at a lower level, but with up and down fluctuations. Since no article was selected in 2006, the annual average number of citations was 0. These results indicate that anti-NMDA encephalitis has received increasing attention from scholars since 2007, and a large number of scholars have published articles in this field. The gradual increase in the number of articles published in recent years indicates that there are still some unresolved problems in the field of anti-NMDA that deserve further research and consideration by scholars.

### Analysis of countries and regions

**Figure 3:**
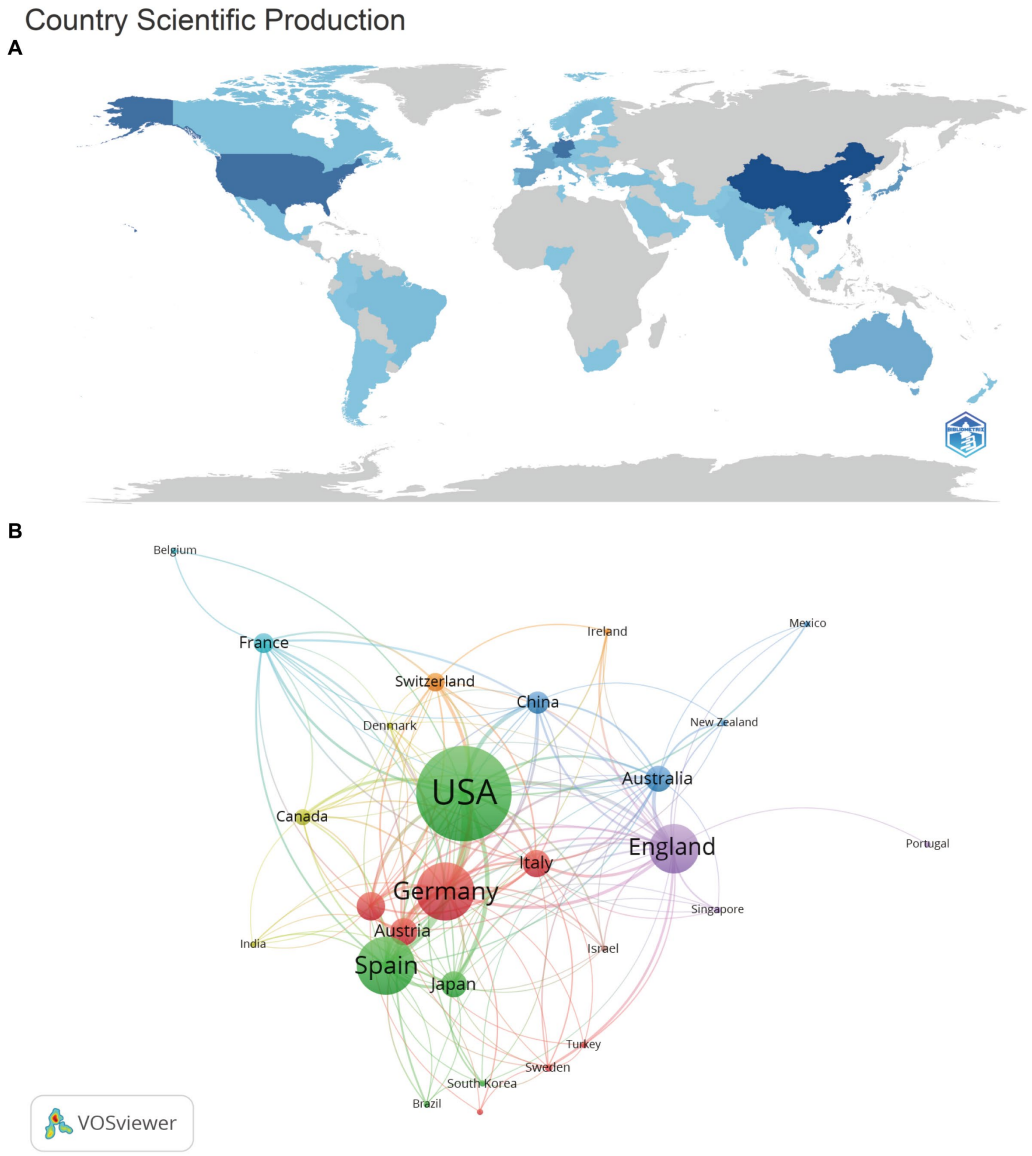
Relationship between publications and countries. **(A)** Country maps of publications. **(B)** Collaborative networks among the source countries of the publications.

We summarized and ranked the top ten countries of origin of the authors of the articles in the material, and calculated the average number of citations per article (NC/NP) for each country for analyzing the quality of their articles (Table 1). As shown in the figure, China is the top country in terms of number of publication (NP), followed by USA (*n* = 274) and Germany (*n* = 117). At the same time, we show the number of publications in each country on a world map (Figure 3A), where darker colors indicate more publications. The map visualizes the data in the table, and there are 68 countries in the world that have published research articles in the field related to anti-NMDA encephalitis, with a larger number of countries that have published only one paper (e.g., Argentina, Estonia, etc.).

**Table 1:** Summary of the top 10 countries in terms of publications.

Rank	Country	NP	NC	NC/NP
1	China	350	3,579	10.23
2	USA	274	20,058	73.20
3	Germany	117	6,881	58.81
4	Japan	107	6,157	57.54
5	England	93	4,933	53.04
6	Spain	75	10,823	144.31
7	Australia	54	2,693	49.87
8	France	43	1,678	39.02
9	Italy	35	684	19.54
10	India	30	443	14.77

The top three countries in NC/NP are Spain (*n* = 144.31), USA (*n* = 73.20) and Germany (*n* = 58.81). China’s NC/NP is in the last place among the top ten countries (*n* = 10.23), far behind the rest of the top ten countries in terms of volume of articles. USA is in the top place in terms of quality of articles and NC/NP. It is noteworthy that Spain ranks sixth in terms of the number of articles sent, but is located in the first place in terms of NC/NP ranking, which is 1.97 times higher than that of USA and 14.11 times higher than that of China. Meanwhile, we set the minimum number of articles per country to 5, obtained 30 countries, and analyzed their mutual cooperation using VOS viewer (Figure 3B). Both the size of each node and the thickness of the connecting line indicate the strength of cooperation between countries. Among them, USA and Spain have the greatest number of cooperation, the strongest relationship, and the thickest connecting line. Moreover, USA is the country with the most cooperative relationships, followed by Germany.

### Analysis of institutions

The top 10 institutions with the highest number of publications in the field related to anti-NMDA encephalitis are shown in Table 2. The University of Pennsylvania from USA is the institution with the highest number of publications (*n* = 81), followed by the University of Barcelona from Spain (*n* = 53). The University of Oxford from England was in third place with 41 relevant articles. The 10 institutions with the highest number of articles are 5 from China and 1 from Spain, however, the average frequency ranking is different from the number of articles. The first place is still the University of Pennsylvania (*n* = 191.52), while the second and third places are changed to the University of Barcelona (*n* = 109.81) from Spain and CharitÉ-Universitätsmedizin Berlin (*n* = 109.81) from Germany, respectively. Universitätsmedizin Berlin from Germany (*n* = 92.56). 5 institutions from China are at the bottom of the list in terms of average citation frequency and lag far behind the rest of the countries on the list. For example, Zhengzhou University is the last place (*n* = 5.65), while the average citation frequency of the first-ranked institution is up to 33.89 times higher than that of it.

**Table 2:** Summary of the top 10 institutions in terms of publications.

Rank	Institution	Country/Region	NP	NC	NC/NP
1	University of Pennsylvania	USA	81	15,513	191.52
2	University of Barcelona	Spain	53	5,820	109.81
3	University of Oxford	England	41	2,435	59.39
4	Capital Medical University	China	40	585	14.63
5	Sichuan University	China	40	633	15.83
6	Sun Yat Sen University	China	31	195	6.29
7	University of Sydney	Australia	26	1,333	51.27
8	Southern Medical University	China	23	231	10.04
9	CharitÉ–Universitätsmedizin Berlin	Germany	20	1853	92.65
10	Zhengzhou University	China	20	113	5.65

### Analysis of authors

We have summarized the number of publications and citation frequency of the top 10 authors with the highest number of publications in Table 3. From the table, it is clear that the author with the highest number of publications is Dalmau Josep (*n* = 69), followed by Vincent Angela (*n* = 37) and Pruess Harald (*n* = 30). The total number of publications by the top 10 authors is 278, which is 23.94% of the total number of articles in the material, with Dalmau Josep contributing another 5.94% of the total number of articles. The top three authors of NC/NP are Titulaer Maarten J (*n* = 230.27), Graus Francesc (*n* = 222.23) and Dalmau Josep (*n* = 214.04). It is worth noting that although Zhou Dong was selected as one of the top 10 authors in terms of publications, his NC/NP value was low at 17.27.

**Table 3:** Summary of the top 10 authors in terms of publications.

Rank	Author	NP	NC	NC/NP
1	Dalmau, Josep	69	14,769	214.04
2	Vincent, Angela	37	3,045	82.30
3	Pruess, Harald	30	1,430	47.67
4	Titulaer, Maarten J.	22	5,066	230.27
5	Graus, Francesc	22	4,889	222.23
6	Tanaka, Keiko	22	380	17.27
7	Dale, Russell C.	21	1,283	61.10
8	Armangue, Thais	20	3,566	178.30
9	Zhou, Dong	19	373	19.63
10	Iizuka, Takahiro	16	2,764	172.75

**Figure 4:**
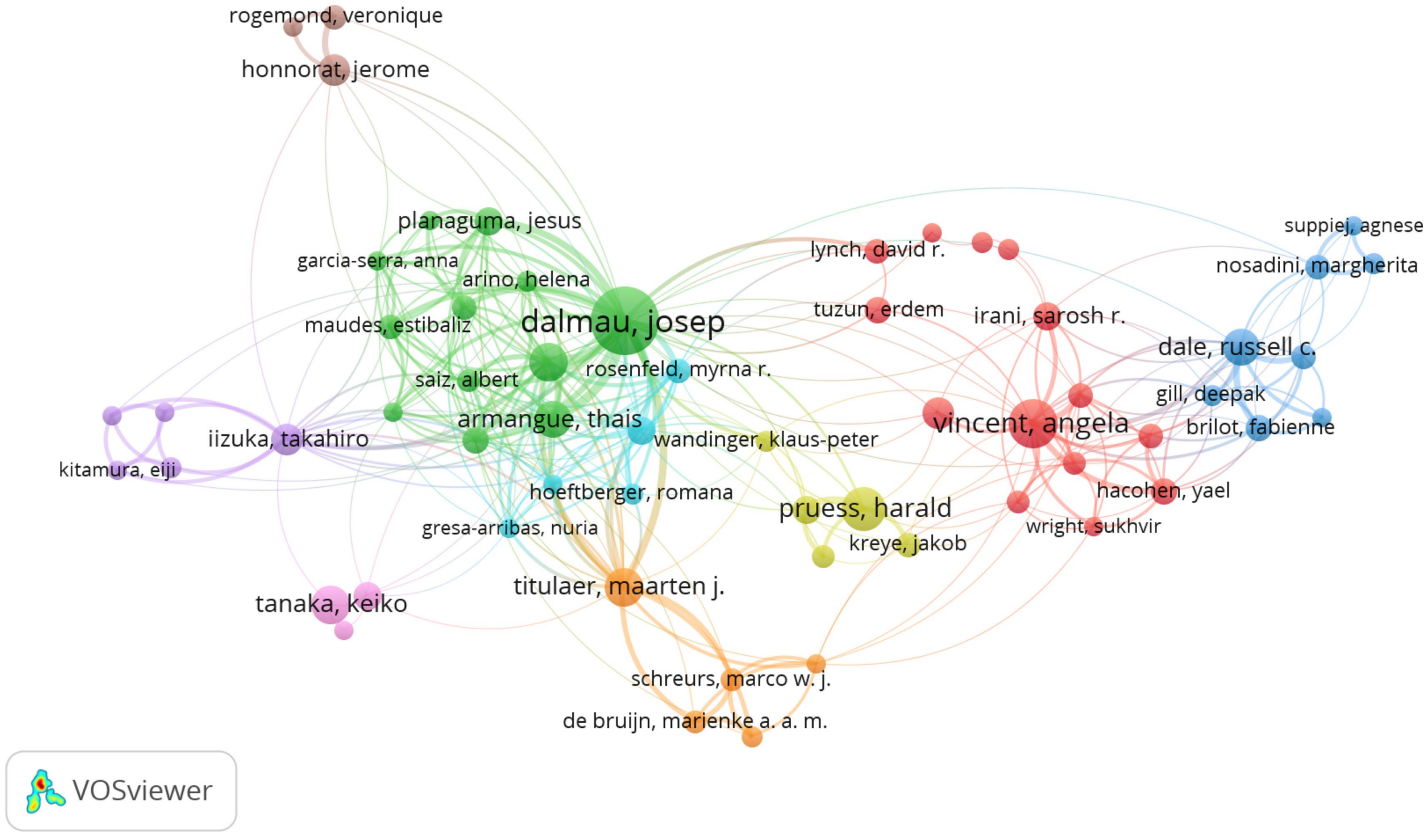
Authors’ collaborative networks.

At the same time, we analyzed the authors’ partnership network using VOS viewer software (Figure 4). The criterion of authors included in the analysis was limited to those who had published at least 6 articles, and 112 authors were obtained for analysis and plotted. The size of the nodes indicates how many articles have been published, and the connecting lines between the nodes indicate the existence of a collaborative relationship between them. Different colors represent different clusters, and it can be found that the blue and cyan clusters are very closely connected, while the blue and brown clusters are relatively distant. As shown in the figure, Dalmau Josep is the author with the widest collaboration and also ranks first in terms of the number of publications. It can be seen that his position in the field of anti-NMDA encephalitis is very important and his contribution is very great, and it can be considered that Dalmau Josep is an important hub for authors who publish in the field of anti-NMDA encephalitis.

### Analysis of journals

The top 10 journals with the highest number of articles published a total of 332 articles, representing 28.59% of all articles in the material (Table 4), with *Frontiers in Neurology* publishing the most articles (*n* = 68, IF = 3.4), followed by *Journal of Neuroimmunology* (*n* = 44, IF = 3.3) and *Frontiers in Immunology* (*n* = 40, IF = 7.3). Notably, despite ranking first in terms of number of articles, *Frontiers in Neurology* ranked third from the bottom of the 10 journals for NC/CP (*n* = 8.71), and *Frontiers in Immunology* ranked at the bottom with 5.30. Similarly, although *Annals of Neurology* ranked 10th with only 18 publications, it ranked first with a high NC/NP (*n* = 247.58).

**Table 4:** Summary of the top 10 journals in terms of publications.

Rank	Journals	NP	NC	NC/NP	IF (2022)	Category quartile
1	*Frontiers in Neurology*	68	592	8.71	3.4	Q2
2	*Journal of Neuroimmunology*	44	627	14.25	3.3	Q3
3	*Frontiers in Immunology*	40	212	5.30	7.3	Q1
4	*BMC Neurology*	30	258	8.60	2.6	Q3
5	*Neurology-Neuroimmunology & Neuroinflammation*	27	781	28.93	8.8	Q1
6	*Pediatric Neurology*	23	325	14.13	3.8	Q2
7	*Neurology*	21	2,548	121.33	10.1	Q1
8	*Journal of Neurology*	21	483	23.00	6	Q1
9	*Journal of Child Neurology*	20	467	23.35	1.9	Q4
10	*Annals of Neurology*	19	4,704	247.58	11.2	Q1

**Figure 5:**
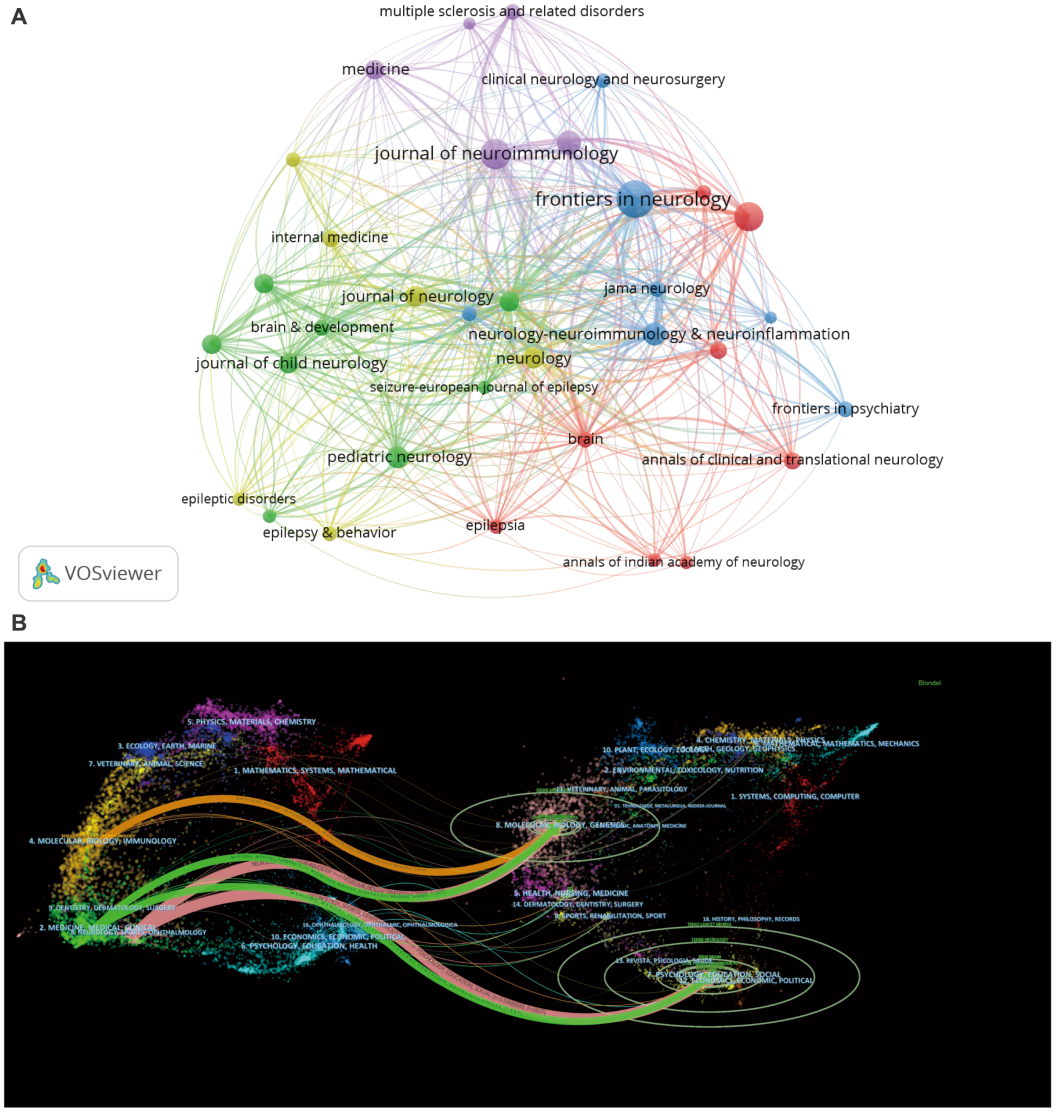
Journal analysis. **(A)** Map of citation relationships between journals. **(B)** Double graphical overlay of journals.

We set the criteria for journals to be included in the analysis to have published at least 8 articles on VOS viewer, and obtained 34 journals for analysis (Figure 5A). The node size represents the number of published articles; the larger the node, the higher the number of published articles. The connecting lines between nodes represent the citation relationship between journals. Different colors represent different clusters. It can be found that all clusters are more closely linked to each other, and only individual journals have relatively few citation relationships compared with other journals.

In the double map overlay of journals (Figure 5B). On the left is the distribution of journals in which the citing literature is located, representing the main disciplines to which the articles in the material belong; on the right is the distribution of journals in which the corresponding cited literature is located, representing the main disciplines in which the articles in the material are cited. The colored paths represent citation pathways. Studies published in Molecular, Biology and Genetics are commonly cited by studies in Molecular, Biology, Immunology, Medicine, Medical, Clinical, Neurology, Sports and Ophthalmology. Citation. Articles published in the disciplines of Psychology, Education, Social, Economics, Economic and Political are often cited by research in Medicine, Medical, Clinical, Neurology, Sports and Ophthalmology research.

### Analysis of highly cited articles

The information related to the 10 most highly cited frequency articles is listed in Table 5, for brevity we have listed only the first author of each article. The table shows that Dalmau Josep contributed 2 highly cited papers. Moreover, the top two cited articles were published in Lancet Neurology (IF = 48, Q1). The first one is written by Dalmau Josep et al. in 2008 published in Lancet Neurology with the title Anti-NMDA-receptor encephalitis: case series and analysis of the effects of antibodies (13). The authors of this article summarized the clinical and immunological characteristics of 100 patients with encephalitis associated with antibodies to the NR1–NR2 isoform of the NMDA receptor and quantified the NMDA receptor clusters to determine the effect of the antibodies on neuronal cultures, and concluded that the patient-specific clinical manifestations were associated with anti-NMDA encephalitis and that the associated antibodies played an important role in anti-NMDA encephalitis. Titulaer Maarten J et al., on the other hand, carried out studies related to the treatment and prognosis of anti-NMDA encephalitis. In 2007 Dalmau Josep et al. published a study linking anti-NMDA encephalitis with ovarian teratoma. Most of the remaining studies were related to pathogenesis and specific clinical manifestations or symptoms. Regardless of the differences in the direction and approach of these studies, they have greatly enriched the research progress and results in the field of anti-NMDA encephalitis.

**Table 5:** Summary of information related to the top 10 cited articles.

Rank	Titles	Authors	Journals	Publish year	Total citations
1	Anti-NMDA-receptor encephalitis: case series and analysis of the effects of antibodies	Dalmau, Josep *et al*.	*Lancet Neurology*	2008	2,124
2	Treatment and prognostic factors for long-term outcome in patients with anti-NMDA receptor encephalitis: an observational cohort study	Titulaer, Maarten J *et al*.	*Lancet Neurology*	2013	1,919
3	Paraneoplastic anti-N-methyl-D-aspartate receptor encephalitis associated with ovarian teratoma	Dalmau, Josep *et al*.	*Annals of Neurology*	2007	1,680
4	Cellular and Synaptic Mechanisms of Anti-NMDA Receptor Encephalitis	Hughes, Ethan G *et al*.	*Journal of Neuroscience*	2010	782
5	Anti-N-Methyl-D-Aspartate Receptor (NMDAR) Encephalitis in Children and Adolescents	Florance, Nicole R *et al*.	*Annals of Neurology*	2009	780
6	N-methyl-d-aspartate antibody encephalitis: temporal progression of clinical and paraclinical observations in a predominantly non-paraneoplastic disorder of both sexes	Irani, Sarosh R *et al*.	*Brain*	2010	757
7	Antibody titres at diagnosis and during follow-up of anti-NMDA receptor encephalitis: a retrospective study	Gresa-Arribas, Nuria *et al*.	*Lancet Neurology*	2014	624
8	The frequency of autoimmune N-methyl-D-aspartate receptor encephalitis surpasses that of individual viral etiologies in young individuals enrolled in the California encephalitis project	Gable, Mary S *et al*.	*Clinical Infectious Diseases*	2012	479
9	Extreme delta brush A unique EEG pattern in adults with anti-NMDA receptor encephalitis	Schmitt, Sarah E *et al*.	*Neurology*	2012	461
10	Immunopathology of autoantibody-associated encephalitides: clues for pathogenesis	Bien, Christian G *et al*.	*Brain*	2012	417

### Analysis of keywords

**Figure 6:**
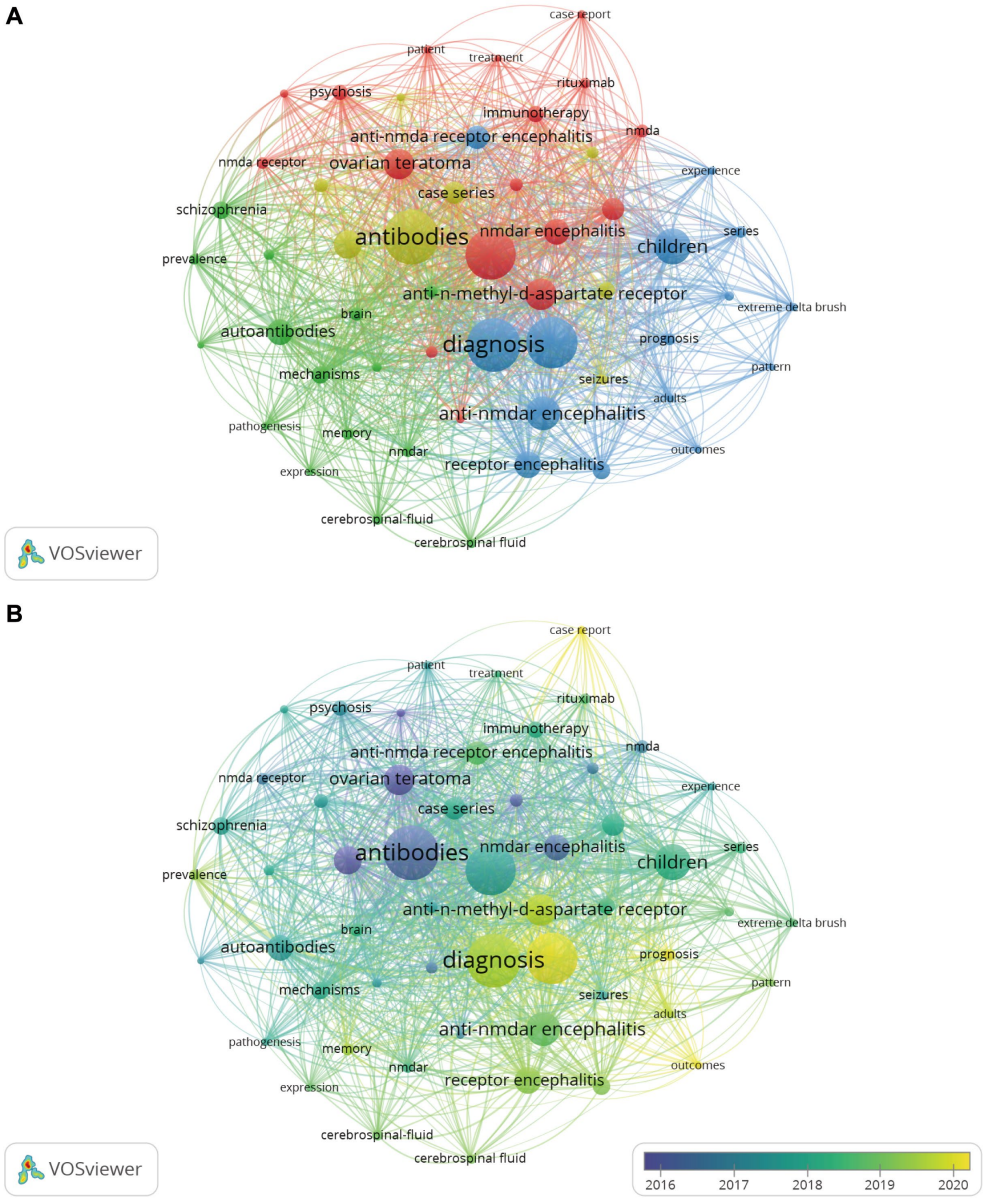
Keyword co-occurrence analysis. **(A)** Keyword co-occurrence network. **(B)** Keyword visualization analysis based on APY. APY, the average publication year.

Keywords are the research topics and core content of an article. In order to explore the research hotspots within the anti-NMDA field, we conducted a keyword analysis (Figure 6). The number of keyword co-occurrences was set to be no less than 25, and 55 more core keywords were obtained, which were categorized into 4 clusters (Figure 6A). Cluster 1 (red, 17) correlates with cases of disease and treatment modalities in the clinic. Cluster 2 (green, 15) is associated with clinical manifestations of anti-NMDA encephalitis. Cluster 3 (blue, 15) is associated with age of onset, and clinical mechanisms. Cluster 4 (yellow, 8) is associated with neuropsychiatric symptoms. Figure 6B shows the relationship between the keywords and time, from blue to yellow representing the keywords appearing farther back in time. For example, “antibodies” is in blue, it means that the keyword appeared earlier, around 2016. The latest keywords are “anti-NMDAR,” “case report” and “encephalitis,” etc., which are closely related to the pathogenesis and individual clinical manifestations. They are closely related to the pathogenesis and individual clinical manifestations and treatment. Moreover, it is worth noting that the keywords with more than 20 occurrences are concentrated in the period from 2016 to 2020.

**Figure 7:**
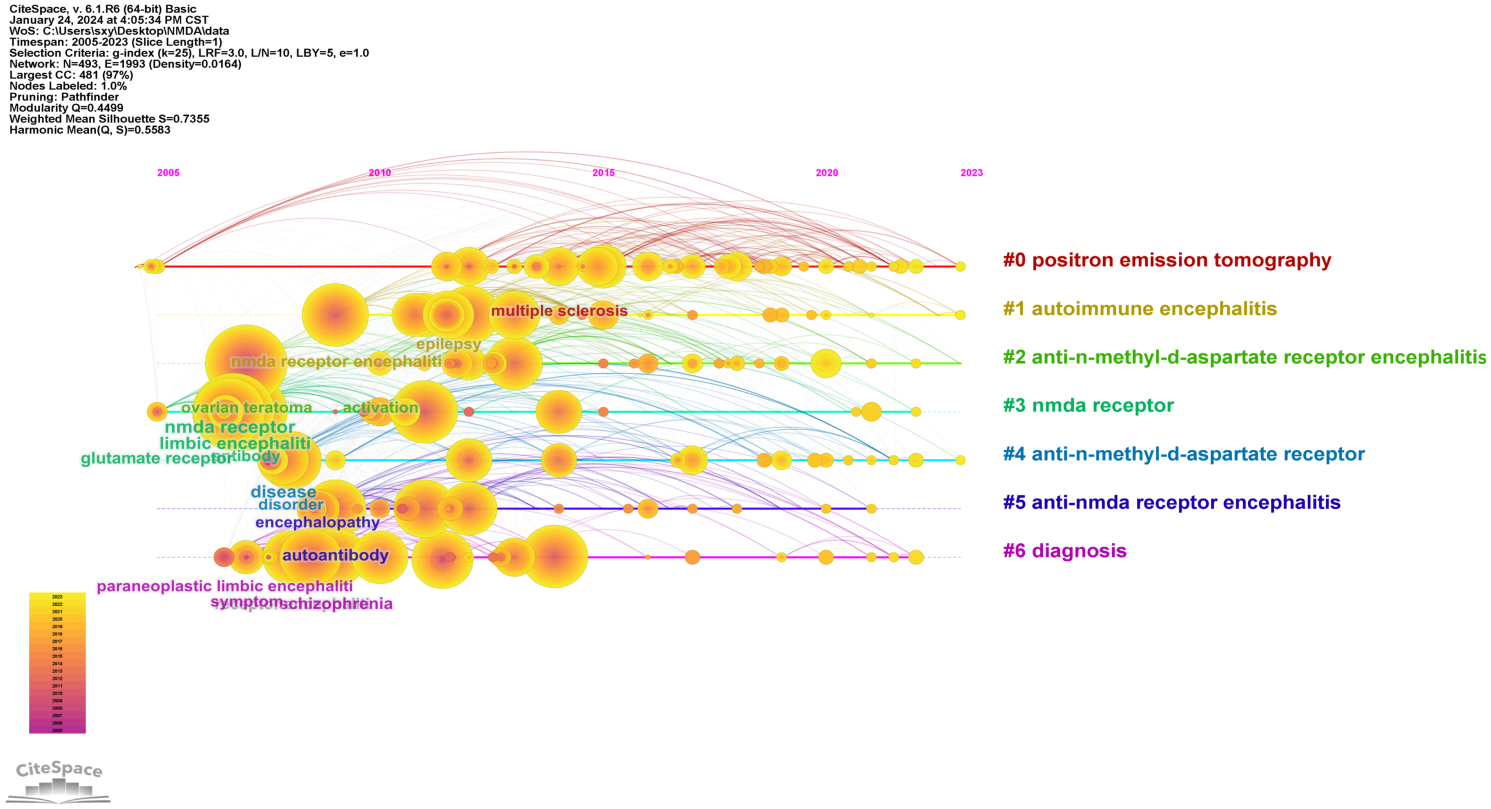
Keyword timeline graph.

Subsequently we used Cite space to analyze the keyword timeline graph (Figure 7). It can be found that the pathogenesis of anti-NMDA encephalitis as well as specific receptors have been a hot topic of research. Diagnostic modalities such as positron emission tomography have also been one of the hot spots of research in recent years.

**Figure 8:**
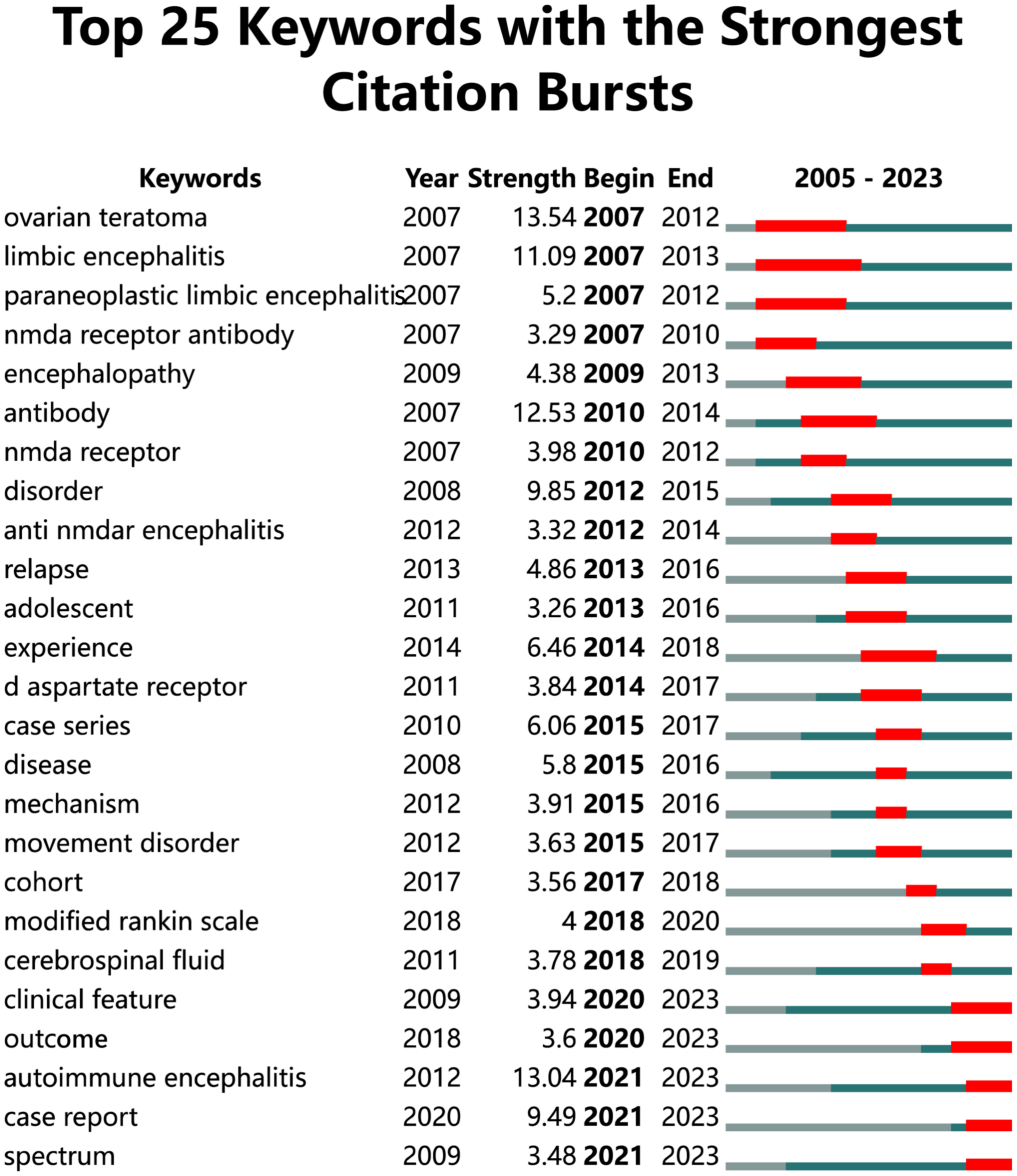
Outbreak word map analysis.

Conducting outburst words can identify the evolutionary trend of research in a certain field from macro to micro and from single to diversified, review what time period the keywords have become hotspots, and even predict which keywords can continue the outburst trend in the future to provide scholars with reference. Therefore, we conducted an exploding word graph analysis (Figure 8), where the blue line indicates the time interval and the red line indicates the citation exploding duration, indicating the change of research hotspots over time. As can be seen from the figure, ovarian teratoma was the word with the strongest outbreak intensity (*n* = 13.54). And limbic encephalitis is the keyword with the longest outbreak, lasting for 6 years. Keywords that have only erupted in recent years, such as “autoimmune encephalitis,” “case report,” and “spectrum,” are more clinically relevant than the earlier outbreaks.

## Discussion

Anti- N-methyl-D-aspartate receptor (NMDAR) encephalitis is an autoimmune-mediated disease characterized by a complex neuropsychiatric syndrome, and an antibody to cerebrospinal fluid (CSF) targeting the GluN1 subunit of NMDAR exists (14, 15), a brain parenchymal inflammation associated with autoimmune-related neurologic dysfunction (16). Anti-NMDA receptor encephalitis was first reported in 2005, when symptoms of psychotic features, memory loss, and altered consciousness were found in four young women with ovarian teratomas (17). In 2007, a study found a new autoantibody in cerebrospinal fluid (CSF) or serum of 12 female patients with an early psychotic This discovery ushered in a new era of diagnostic medicine (18). The etiology of anti-NMDA encephalitis is, for the moment, uncertain, but some studies suggest that it may be able to be triggered by viruses, vaccines or tumors (19, 20). IgG antibodies targeting the GluN1 subunit of the NMDA receptor (NMDAR) are key mediators in the pathogenesis of the disease (21). Antibody-mediated NMDAR dysfunction leads to severe psychiatric symptoms, including memory loss (22), dyskinesia (23), psychotic symptoms (24), drowsiness, seizures (25), impaired consciousness, and even coma in severe cases. Early recognition and rapid activation of a range of immunotherapies may be able to improve the prognosis of patients with anti-NMDA encephalitis. Clinical presentation, cerebrospinal fluid examination, neuroimaging, and electroencephalography can be used for diagnostic typing of encephalitis (26), and are important modalities for guiding the clinical approach to treatment and achieving a favorable outcome for patients. As research progresses, relevant animal models have begun to reveal underlying pathogenic mechanisms and will lead to the development of new therapies in addition to immunotherapy (3). This study was analyzed by bibliometrics to understand the research directions and hotspots of anti-NMDA encephalitis.

The results suggest that the number of articles published in the field of anti-NMDA encephalitis is increasing from 2005 to 2022, which indicates that anti-NMDA encephalitis is becoming more and more popular and widely researched in the academic world. 2023 has a lower number of articles, probably because some articles have not been included in Web of Science. However, the number of publications in 2023 is still large relative to the beginning of the 21st century, so it can be assumed that anti-NMDA encephalitis continues to receive extensive attention from researchers. The frequency of citations is an important indicator of the attention and recognition of articles in this field (27). A search in 2007, the year with the highest average annual citation frequency, revealed that only two articles were published in 2007: Paraneoplastic anti-N-methyl-D-aspartate receptor encephalitis associated with ovarian teratoma (28) (NC = 1,680) and A patient with encephalitis associated with NMDA receptor antibodies (29) (NC = 199). They all had relatively high citation frequencies, which may explain the high average annual citation frequency in 2007. In 2008, we published 5 articles, although 2 highly cited papers were published: Anti-NMDA-receptor encephalitis: case series and analysis of the effects of antibodies (13) (NC = 2,124) and Anti-NMDA receptor encephalitis in Japan (23) (NC = 364), the remaining 3 papers were not highly cited, which may be one of the reasons for the low average citation frequency in 2008.

China has carried out a lot of research in the field of anti-NMDA encephalitis in the past 18 years, and five institutions from China have been selected in the list of top 10 institutions in terms of the number of articles published, but the number of no self-citation (NC) is not high, and the NC/NP is in the last position in the top 10, which is much lower than that of other countries. This indicates that the number of articles published in China is already in an excellent state, and it should focus on the quality of the articles. China has a long way to go in publishing more quality and influential articles. Of course, this may also be related to the late development of research related to anti-NMDA encephalitis in China. We found that China published its first anti-NMDA-related article only in 2010, and then the number of articles published has been growing explosively every year. Spain ranked 6th in terms of the number of articles published, with 75 articles in the field of anti-NMDA encephalitis, but with the highest NC/NP, suggesting that Spain has had a more prominent contribution to the field in the past 18 years, and has published at least one highly cited article in the field. Through the search, we were surprised to find that the most prolific author, Dalmau Josep, is affiliated with the University of Barcelona in Spain and has two highly cited articles, and he is also the first author to systematically study anti-NMDA encephalitis, which may be a major reason why Spain has such excellent NC/NP data. USA has an excellent publication volume and NC/NP, which may be related to the fact that USA has a strong research team and sufficient research funding. Among all the countries, USA and Spain have the closest collaboration, so the two countries are at the forefront of research in this field, which is well represented in the field of anti-NMDA encephalitis. It shows that mutual cooperation can greatly contribute to academic progress, and the ability to conduct more research with greater impact in a particular area is a good way to progress together.

The top 10 journals in the NP rankings all have high impact factors. It indicates that these journals are more interested in articles in the field of anti-NMDA encephalitis and have some influence in the field. Authors conducting research in this field may consider publishing their studies in these journals. Three of the top 10 cited articles were published in Lancet Neurology, which has a high impact factor, indicating that it is a top journal in the field of anti-NMDA encephalitis, and authors who have made significant breakthroughs in anti-NMDA encephalitis research may consider publishing their studies in this journal.

The top 10 cited articles show that research in the field of anti-NMDA encephalitis has emphasized the clinical manifestations, diagnosis and treatment of the disease. And there is also a strong emphasis on diseases associated with the development of anti-NMDA encephalitis, such as ovarian teratoma (30) and herpes simplex (31). Scholars may consider conducting research to discover how anti-NMDA encephalitis combines with other diseases, or to explore a new treatment with a better prognosis, to propose a faster and more accurate diagnostic method is also a major direction for research. Since the cause of anti-NMDA encephalitis is not well understood, research into the etiology of the disease could be conducted, which could also provide clues to the diagnosis of the disease.

Keyword clustering mapping showed that studies in the field of anti-NMDA encephalitis were mainly centered on its definition, pathogenesis, clinical manifestations, diagnostic indicators, therapeutic modalities, and case reports, and the results confirmed that anti-NMDA encephalitis is an autoimmune-mediated disease manifesting as a complex neuropsychiatric syndrome, which results in a wide range of neurological symptoms, including psychiatric symptoms, movement disorders, memory disorders (14). And according to the clustering results, for cluster 2 we explored the specific clinical manifestations of anti-NMDA encephalitis, suggesting that patients with anti-NMDA receptor encephalitis present a characteristic stage-by-stage clinical course, and according to the course curve of classical anti-NMDAR encephalitis drawn by Kayser and Dalmau (32), we found that patients often have an antecedent infection 2 weeks before the onset of the disease, which leads to the development of certain antecedent symptoms, mainly viral cold symptoms; 1–2 weeks of progression of psychiatric symptoms, such as hallucinations, mania, delusions, anxiety, insomnia; weeks to months of neurological complications: disorders of consciousness, central hypoventilation, and even coma, often accompanied by dyskinesia and seizures; and months to years of neurological symptoms: executive functions, hypoparathyroidism and hyperventilation. Long-term deficits such as executive dysfunction, impulsivity and sleep disturbances are manifested over a period of months to years, and the critical but relatively reversible nature of the disease is an important feature. It was even found that the course of recovery from the disease was consistent with the course of disease progression, i.e., the first symptoms to appear disappeared last. However, it is worth noting that there may be some differences between children and adults (33), and we believe that the most common clinical manifestations of anti-NMDA receptor encephalitis in children are epilepsy, movement disorders, and personality changes, as well as the incidence of comorbid tumors in pediatric patients is lower than in adults (17). The younger the age, the lower the likelihood of tumor development, suggesting that the pathogenesis may differ between children and adults (34). Research into potential clinical treatment modalities for anti-NMDA encephalitis has been an outbreak hotspot in recent years. The treatment of anti-NMDA receptor encephalitis mainly relies on searching for the relevant causes and treating the causes as much as possible, including receiving immunotherapy, supportive therapy, physical therapy, prevention of secondary infections and symptomatic treatment as early as possible, which can be categorized into first-line and second-line treatment. Plasma exchange, intravenous infusion of gamma globulin and steroid hormones are the first line of immunotherapy, and most patients respond well to the first line of treatment, and removal of tumors in patients with combined tumors can improve the efficacy and shorten the course of treatment (35). Second-line drug therapy, including rituximab and cyclophosphamide, can often be considered when patients with anti-NMDA receptor encephalitis do not respond significantly to first-line drug therapy, but overall immunotherapy for anti-NMDA receptor encephalitis remains the main effective approach at present.

This study focuses on a new type of autoimmune encephalitis anti-NMDA encephalitis. And the discovery of NMDA receptor has opened up the research field of anti-NMDA encephalitis which reveals the pathogenesis of anti-NMDA encephalitis and also provides an important theoretical basis for the development of drugs targeting NMDA receptor which brings a new hope for the treatment of patients. Among the six clusters in the keyword timeline graph two are related to anti-NMDA encephalitis and two are related to anti-NMDA receptor just using different forms to convey the same meaning. In addition through the timeline we found that in recent years the research on anti-NMDA encephalitis has gradually shifted from the previous basic research to focus on the clinical direction including its clinical features outcomes and related case reports but there are still fewer research articles suggesting that future scholars can focus on the clinical aspect of the research to explore new manifestations and characteristics. In the outbreak word map the keywords for outbreaks in the last 3 years are “Modified Rankin Scale” “cerebrospinal fluid” “clinical feature” “outcome” “autoimmune encephalitis” “case report” “spectrum” which are very closely related to each other. Overall they are related to the etiology diagnosis clinical features prognosis and genre of the article on anti-NMDA encephalitis, respectively. The Anti-NMDA receptor encephalitis is a rare autoimmune disease with clinical manifestations often resembling psychiatric disorders (e.g., convulsions disorders of consciousness etc.) and thus its clinical manifestations are often masked by psychosis making accurate diagnosis a challenge for clinicians clinical presentation and characteristics of this disease have become a recent focus. Because of the lack of accurate clinical features researchers are exploring various methods for more precise diagnosis of anti-NMDA receptor encephalitis. Currently the mainstream clinical diagnostic approach involves testing for the presence of anti-NMDA receptor antibodies in the patient’s cerebrospinal fluid or serum aiding in distinguishing individuals truly affected by anti-NMDA receptor encephalitis from those with psychiatric-like symptoms (36). However as antibody detection results and treatment responses are not available at disease onset this method may potentially delay patient management (26). Positron Emission Tomography (PET) has also been utilized for the diagnosis of anti-NMDA receptor encephalitis assessing functional rather than structural abnormalities. PET scans show overall reduced brain metabolism and focal hypermetabolic lesions in the right cerebellar cortex but a combination with antibody detection is still necessary for diagnosis (37, 38). Conventional tests (such as EEG) and empiric clinical diagnoses lack specificity and evidence highlighting the need for faster and more accurate diagnostic methods in the future (39). Of course these keywords are connected to much more than that. In recent years the Modified Rankin Scale (mRS) has gained attention as an important assessment tool (40). It assigns grade scores evaluates independent living capabilities and uses walking ability as a specific scoring criterion to assess patient prognosis. The mRS is widely used in clinical research particularly in case reports to objectively document patient functional status and quality of life providing crucial data support for research (2, 41). The latest emerging term “spectrum” in the burst word graph refers to the disease etiology spectrum of anti-NMDA receptor encephalitis. Known triggers include ovarian teratomas herpes simplex and other tumors or viral infections but the majority of patients with anti-NMDA receptor encephalitis have unidentified triggers posing significant challenges for prevention and diagnosis (3, 42). Expanding the disease spectrum can guide clinical practitioners enhance diagnostic speed and accuracy and shed light on potential causes of anti-NMDA receptor encephalitis prompting researchers to analyze and study in this direction. Therefore expanding the disease spectrum through individual or multiple specific etiology case reports is crucial (43). Case reports are an excellent way to summarize and document specific cases and help researchers identify new clinical features of the disease. In turn the use of the case report format and the mRS assessment scale to summarize and analyze patient prognosis provides an objective reference for the diagnosis and treatment of anti-NMDA encephalitis and lays the foundation for continuous optimization of the diagnosis and treatment of the disease. Overall anti-NMDA encephalitis which was only formally established as a separate disease entity in 2007 has become more comprehensively understood over the past 16 years but further multicenter and expanded sample studies are needed to explore and refine therapeutic approaches and to search for more specific clinical manifestations and a broader range of disease-related etiologies. Evaluation of the patient’s prognosis is also an integral part of the process which will directly guide the implementation of clinical treatment programs. Intensive research in these areas will likely greatly improve the prognosis of patients and allow them to have a better quality of life.

This study is bibliometric study to aggregate, summarize, and analyze relevant articles in the field of anti-NMDA encephalitis from 2005 to 2023. With these results, we provide scholars with information on research hotspots and future research trends. Scholars can conduct relevant research based on these results and select suitable journals for their submissions. It also helps scholars to choose partners to conduct research together. At the same time, it allows each country, organization, etc. to understand the shortcomings of their published articles and the direction of future efforts.

Of course, this study has some shortcomings. First, our study only included English articles and proceeding papers, and classic articles in other languages and types could not be included. Second, articles in the field of anti-NMDA encephalitis are constantly updated, and our study can only provide a historical perspective. Third, data from other important databases such as PubMed, Scopus were excluded, which may make us lose some classic articles that are not available in Web of Science database.

## Conclusion

Bibliometric analysis shows that anti-NMDA encephalitis is a hot disease for research in recent years, which is in a rapid development stage. Spain and USA are the most influential countries, which have made significant contributions to the development of the anti-NMDA encephalitis field. China has a huge number of studies, but the quality needs to be improved. And the remaining countries such as those in Africa and South America should strengthen the research efforts in the field of anti-NMDA encephalitis. A broader and clearer etiological spectrum of anti-NMDA encephalitis, rapid and highly positive diagnosis, clinical characterization, and continuous updating and refinement of therapeutic modalities by comparative analysis of patients’ prognosis are hot topics for future research.

## Data availability statement

The raw data supporting the conclusions of this article will be made available by the authors, without undue reservation.

## Author contributions

XS: Data curation, Formal analysis, Project administration, Resources, Supervision, Validation, Writing – original draft. ZL: Data curation, Formal analysis, Writing – original draft. DH: Software, Visualization, Writing – original draft. JL: Software, Visualization, Writing – original draft. LX: Software, Visualization, Writing – original draft. TL: Conceptualization, Methodology, Writing – review & editing. KZ: Conceptualization, Methodology, Writing – review & editing.

## References

[1] Seery N Butzkueven H O'Brien TJ Monif M. Contemporary advances in anti-NMDAR antibody (ab)-mediated encephalitis. Autoimmun Rev. (2022) 21:103057. doi: 10.1016/j.autrev.2022.103057, PMID: 35092831

[2] Ratuszny D Skripuletz T Wegner F Gross M Falk C Jacobs R . Case report: Daratumumab in a patient with severe refractory anti-NMDA receptor encephalitis. Front Neurol. (2020) 11:5. doi: 10.3389/fneur.2020.602102 33414761 PMC7782967

[3] Dalmau J Armangué T Planagumà J Radosevic M Mannara F Leypoldt F . An update on anti-NMDA receptor encephalitis for neurologists and psychiatrists: mechanisms and models. Lancet Neurol. (2019) 18:1045–57. doi: 10.1016/S1474-4422(19)30244-3, PMID: 31326280

[4] Maneta E Garcia G. Psychiatric manifestations of anti-NMDA receptor encephalitis: neurobiological underpinnings and differential diagnostic implications. Psychosomatics. (2014) 55:37–44. doi: 10.1016/j.psym.2013.06.002, PMID: 23932531

[5] Breese EH Dalmau J Lennon VA Apiwattanakul M Sokol DK. Anti-N-methyl-D-aspartate receptor encephalitis: early treatment is beneficial. Pediatr Neurol. (2010) 42:213–4. doi: 10.1016/j.pediatrneurol.2009.10.003, PMID: 20159432

[6] Mukherjee D Lim WM Kumar S Donthu N. Guidelines for advancing theory and practice through bibliometric research. J Bus Res. (2022) 148:101–15. doi: 10.1016/j.jbusres.2022.04.042

[7] Chen CM. Searching for intellectual turning points: progressive knowledge domain visualization. Proc Natl Acad Sci USA. (2004) 101:5303–10. doi: 10.1073/pnas.0307513100, PMID: 14724295 PMC387312

[8] van Eck NJ Waltman L. Citation-based clustering of publications using CitNetExplorer and VOSviewer. Scientometrics. (2017) 111:1053–70. doi: 10.1007/s11192-017-2300-7, PMID: 28490825 PMC5400793

[9] Aria M Cuccurullo C. bibliometrix: an R-tool for comprehensive science mapping analysis. J Informet. (2017) 11:959–75. doi: 10.1016/j.joi.2017.08.007

[10] Donthu N Kumar S Mukherjee D Pandey N Lim WM. How to conduct a bibliometric analysis: an overview and guidelines. J Bus Res. (2021) 133:285–96. doi: 10.1016/j.jbusres.2021.04.070

[11] Garfield E. The history and meaning of the journal impact factor. JAMA. (2006) 295:90–3. doi: 10.1001/jama.295.1.90, PMID: 16391221

[12] Roldan-Valadez E Salazar-Ruiz SY Ibarra-Contreras R Rios C. Current concepts on bibliometrics: a brief review about impact factor, Eigenfactor score, CiteScore, SCImago journal rank, source-normalised impact per paper, H-index, and alternative metrics. Ir J Med Sci. (2019) 188:939–51. doi: 10.1007/s11845-018-1936-5, PMID: 30511320

[13] Dalmau J Gleichman AJ Hughes EG Rossi JE Peng XY Lai MZ . Anti-NMDA-receptor encephalitis: case series and analysis of the effects of antibodies. Lancet Neurol. (2008) 7:1091–8. doi: 10.1016/S1474-4422(08)70224-2, PMID: 18851928 PMC2607118

[14] Liu P Yan H Li HZ Zhang CH Li YF. Overlapping anti-NMDAR encephalitis and multiple sclerosis: a case report and literature review. Front Immunol. (2023) 14:7. doi: 10.3389/fimmu.2023.1088801 PMC9923169 36793718

[15] Wu CY Wu JD Chen CC. The Association of Ovarian Teratoma and Anti-N-methyl-D-aspartate receptor encephalitis: an updated integrative review. Int J Mol Sci. (2021) 22:22. doi: 10.3390/ijms222010911 PMC8535897 34681570

[16] Venkatesan A Tunkel AR Bloch KC Lauring AS Sejvar J Bitnun A . Case definitions, diagnostic algorithms, and priorities in encephalitis: consensus statement of the international encephalitis consortium. Clin Infect Dis. (2013) 57:1114–28. doi: 10.1093/cid/cit458, PMID: 23861361 PMC3783060

[17] Titulaer MJ McCracken L Gabilondo I Armangué T Glaser C Iizuka T . Treatment and prognostic factors for long-term outcome in patients with anti-NMDA receptor encephalitis: an observational cohort study. Lancet Neurol. (2013) 12:157–65. doi: 10.1016/S1474-4422(12)70310-1, PMID: 23290630 PMC3563251

[18] Fischer CE Golas AC Schweizer TA Munoz DG Ismail Z Qian WN . Anti N-methyl-D-aspartate receptor encephalitis: a game-changer? Expert Rev Neurother. (2016) 16:849–59. doi: 10.1080/14737175.2016.1184088, PMID: 27123777

[19] Wang SY. Anti-NMDA receptor encephalitis, vaccination and virus. Curr Pharm Des. (2019) 25:4579–88. doi: 10.2174/1381612825666191210155059 31820697

[20] Wang HY. Anti-NMDA receptor encephalitis and vaccination. Int J Mol Sci. (2017) 18:9. doi: 10.3390/ijms18010193 PMC5297824 28106787

[21] Nan D Zhang Y Han JM Jin T. Clinical features and management of coexisting anti-N-methyl-D-aspartate receptor encephalitis and myelin oligodendrocyte glycoprotein antibody-associated encephalomyelitis: a case report and review of the literature. Neurol Sci. (2021) 42:847–55. doi: 10.1007/s10072-020-04942-0, PMID: 33409829

[22] Endres D Rauer S Kern W Venhoff N Maier SJ Runge K . Psychiatric presentation of anti-NMDA receptor encephalitis. Front Neurol. (2019) 10:9. doi: 10.3389/fneur.2019.01086 31749755 PMC6848057

[23] Iizuka T Sakai F Ide T Monzen T Yoshii S Iigaya M . Anti-NMDA receptor encephalitis in Japan. Neurology. (2008) 70:504–11. doi: 10.1212/01.wnl.0000278388.90370.c3, PMID: 17898324 PMC2586938

[24] Najjar S Pearlman DM Alper K Najjar A Devinsky O. Neuroinflammation and psychiatric illness. J Neuroinflamm. (2013) 10:24. doi: 10.1186/1742-2094-10-43 PMC3626880 23547920

[25] Steriade C Britton J Dale RC Gadoth A Irani SR Linnoila J . Acute symptomatic seizures secondary to autoimmune encephalitis and autoimmune-associated epilepsy: conceptual definitions. Epilepsia. (2020) 61:1341–51. doi: 10.1111/epi.16571, PMID: 32544279

[26] Graus F Titulaer MJ Balu R Benseler S Bien CG Cellucci T . A clinical approach to diagnosis of autoimmune encephalitis. Lancet Neurol. (2016) 15:391–404. doi: 10.1016/S1474-4422(15)00401-9, PMID: 26906964 PMC5066574

[27] Bornmann L Daniel HD. What do citation counts measure? A review of studies on citing behavior. J Doc. (2008) 64:45–80. doi: 10.1108/00220410810844150

[28] Dalmau J Tuzun E Wu HY Masjuan J Rossi JE Voloschin A . Paraneoplastic anti-N-methyl-D-aspartate receptor encephalitis associated with ovarian teratoma. Ann Neurol. (2007) 61:25–36. doi: 10.1002/ana.21050, PMID: 17262855 PMC2430743

[29] Sansing LH Tüzün E Ko MW Baccon J Lynch DR Dalmau J. A patient with encephalitis associated with NMDA receptor antibodies. Nat Clin Pract Neurol. (2007) 3:291–6. doi: 10.1038/ncpneuro0493, PMID: 17479076 PMC1936221

[30] Chefdeville A Treilleux I Mayeur ME Couillault C Picard G Bost C . Immunopathological characterization of ovarian teratomas associated with anti-N-methyl-D-aspartate receptor encephalitis. Acta Neuropathol Commun. (2019) 7:11. doi: 10.1186/s40478-019-0693-7 PMC6410529 30857565

[31] DeSena A Graves D Warnack W Greenberg BM. Herpes simplex encephalitis as a potential cause of anti-N-methyl-D-aspartate receptor antibody encephalitis report of 2 cases. JAMA Neurol. (2014) 71:344–6. doi: 10.1001/jamaneurol.2013.4580, PMID: 24473671

[32] Kayser MS Dalmau J. Anti-NMDA receptor encephalitis in psychiatry. Curr Psychiatr Rev. (2011) 7:189–93. doi: 10.2174/157340011797183184, PMID: 24729779 PMC3983958

[33] Zekeridou A Karantoni E Viaccoz A Ducray F Gitiaux C Villega F . Treatment and outcome of children and adolescents with N-methyl-D-aspartate receptor encephalitis. J Neurol. (2015) 262:1859–66. doi: 10.1007/s00415-015-7781-9 25987208

[34] Luca N Daengsuwan T Dalmau J Jones K deVeber G Kobayashi J . Anti-N-methyl-D-aspartate receptor encephalitis: a newly recognized inflammatory brain disease in children. Arthritis Rheum. (2011) 63:2516–22. doi: 10.1002/art.30437, PMID: 21547896 PMC3703928

[35] Dalmau J Lancaster E Martinez-Hernandez E Rosenfeld MR Balice-Gordon R. Clinical experience and laboratory investigations in patients with anti-NMDAR encephalitis. Lancet Neurol. (2011) 10:63–74. doi: 10.1016/S1474-4422(10)70253-2, PMID: 21163445 PMC3158385

[36] Giri YR Korie I Hashmi S Parrill A Ayed N. Anti-NMDA receptor encephalitis masquerades as psychosis: a case report. J Psychiatr Pract. (2022) 28:72–7. doi: 10.1097/PRA.0000000000000603, PMID: 34989349

[37] Bacchi S Franke K Wewegama D Needham E Patel S Menon D. Magnetic resonance imaging and positron emission tomography in anti-NMDA receptor encephalitis: a systematic review. J Clin Neurosci. (2018) 52:54–9. doi: 10.1016/j.jocn.2018.03.026, PMID: 29605275

[38] Maqbool M Oleske DA Huq AHM Salman BA Khodabakhsh K Chugani HT. Novel FDG-PET findings in anti-NMDA receptor encephalitis: a case based report. J Child Neurol. (2011) 26:1325–8. doi: 10.1177/0883073811405199, PMID: 21596699

[39] Schmitt SE Pargeon K Frechette ES Hirsch LJ Dalmau J Friedman D. Extreme delta brush a unique EEG pattern in adults with anti-NMDA receptor encephalitis. Neurology. (2012) 79:1094–100. doi: 10.1212/WNL.0b013e3182698cd8, PMID: 22933737 PMC3525298

[40] Quinn TJ Dawson J Walters MR Lees KR. Reliability of the modified Rankin scale a systematic review. Stroke. (2009) 40:3393–5. doi: 10.1161/STROKEAHA.109.557256 19679846

[41] Shim Y Kim SY Kim H Hwang H Chae JH Choi J . Clinical outcomes of pediatric anti-NMDA receptor encephalitis. Eur J Paediatr Neurol. (2020) 29:87–91. doi: 10.1016/j.ejpn.2020.10.001 33046392

[42] Venkatesan A Benavides DR. Autoimmune encephalitis and its relation to infection. Curr Neurol Neurosci Rep. (2015) 15:11. doi: 10.1007/s11910-015-0529-1 25637289

[43] Fang F Wang Y Xu W. Anti-N-methyl-D-aspartate receptor encephalitis associated with intracranial cryptococcal infection: a case report and 2-year follow-up. J Neuroimmunol. (2021) 353:577502. doi: 10.1016/j.jneuroim.2021.577502 33548619

